# Combined Locally Enhanced Electric Field Treatment and Copper for Effective Disinfection in a Circulating Water System

**DOI:** 10.1021/acsestengg.5c01041

**Published:** 2026-03-05

**Authors:** Feiyang Mo, Wei Wang, Lavine M. Chuol, Mourin Jarin, James Willie Corley, Xing Xie

**Affiliations:** † School of Civil and Environmental Engineering, 1372 Georgia Institute of Technology, 311 Ferst Drive, Atlanta, Georgia 30332, United States; ‡ 5225 Tift College of Education, Mercer University, 3001 Mercer University Drive, Atlanta, Georgia 30341, United States

**Keywords:** Recreational water disinfection, circulating system, chlorine-free, copper ionization, raw water

## Abstract

Public concerns have increased regarding disinfection byproducts and respiratory health effects associated with chlorination in circulating recreational water environments such as swimming pools and hot tubs. Locally enhanced electric field treatment combined with copper (LEEFT-Cu) has emerged as a promising chlorine-free disinfection technology. However, its performance in circulating systems remains largely unexplored. In this study, a 10 L circulating system incorporating a LEEFT-Cu device was developed to evaluate its in-line disinfection performance. The effects of current and flow rate on microbial inactivation and copper accumulation were systematically investigated. The results revealed that current influenced both copper accumulation and bacterial inactivation in the reservoir, whereas flow rate primarily affected bacterial inactivation. Under the optimal condition (*i.e.*, 80 mL/min, 2 mA), the LEEFT-Cu system achieved a 4.5-log reduction in bacterial concentration within 4 h, which is over 3 logs higher than CuSO_4_ dosing at an equivalent copper concentration. The system operated at an ultralow power of approximately 2.5 × 10^–3^ W and demonstrated effective performance with real water samples. These findings highlight the strong potential of LEEFT-Cu as an energy-efficient, chlorine-free disinfection strategy for circulating recreational water systems.

## Graphical abstract

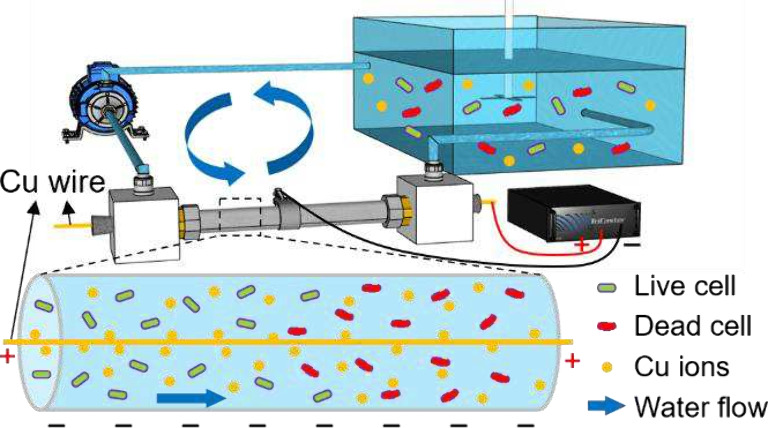

## 1 Introduction

Recreational water environments, including swimming pools, hot tubs, and natural bathing areas, pose potential risks for the transmission of waterborne pathogens.^1^ Chlorination remains the most widely used disinfection method due to its antimicrobial efficacy, residual inactivation capacity, and cost-effectiveness.^2^ However, chlorine reacts with natural organic matter (NOM) in water to produce harmful disinfection byproducts (DBPs), such as chloroform and trihalomethanes.^2^ The formation of DBPs is particularly pronounced in pools and spas, where higher chlorine doses are required to compensate for rapid chlorine decay caused by elevated water temperatures and heavy organic load.^3,4^ Moreover, human inputs (*e.g.*, hair, saliva, sweat, and urine) and additives commonly used in hot tubs (*e.g.*, citric acid and glycerol) further increase the organic load, thereby promoting DBP formation.^4–6^ In addition to DBPs, public concern has grown regarding the respiratory health effects associated with indoor pools and spas.^7,8^ The characteristic “chlorine smell” often reported in these environments is primarily due to chloramines (*e.g.*, NH_2_Cl, NHCl_2_, and NCl_3_), which are formed by the reactions between hypochlorite and nitrogen-containing compounds from swimmers and bathers.^9,10^ Chloramines readily volatilize into the air, and their inhalation has been linked to increased risks of respiratory irritation, particularly among avid swimmers, pool employees, and adolescents.^10–12^ These issues underscore the need for alternative or supplementary disinfection strategies to chlorination in recreational water environments. Existing alternatives include electrochemical salt systems, ultraviolet irradiation (UV), ozonation, and antimicrobial metal ionization (Cu/Ag).^6,13^ However, these approaches are typically used as adjuncts to reduce chlorine demand rather than as complete replacements for chlorination.

To address the limitations of chlorination and other chemical-based disinfectants, locally enhanced electric field treatment (LEEFT) has recently emerged as a promising chlorine-free alternative.^14^ LEEFT offers several advantages, including high inactivation efficiency, low energy consumption, and minimal side reactions.^15^ The technique employs low voltages to locally intensify the electric field near the electrode surfaces, inducing electropermeabilization and/or electroporation of cell membranes.^16^ As a primarily physical process, LEEFT does not alter the physicochemical properties of water and effectively eliminates the risk of DBP formation.^14^ However, the lack of a residual disinfectant provided by LEEFT makes its sole application challenging in high-demand water bodies like pools and spas. Given the intrinsic antimicrobial properties of copper, our group has explored to combine the LEEFT with copper disinfection (LEEFT-Cu) in recent years.^17–19^ Previous studies have demonstrated that bacteria become more susceptible to copper after LEEFT due to increased cell membrane permeability.^20^ The synergistic effects of the electric field and copper enable effective disinfection at low voltages (<3 V) and low copper concentrations (<1 mg/L).^17^ In addition, the intrinsic antimicrobial property of copper provides residual disinfection, offering an advantage over other disinfection technologies such as UV and ozonation.

Despite these benefits, most existing LEEFT-Cu studies have focused on plug-flow systems for point-of-use applications (Table S1), where water flows unidirectionally without recirculation.^17–19,21,22^ In contrast, recreational water environments rely on continuous circulation to maintain water quality. Compared with plug-flow systems, circulating systems introduce unique challenges. First, the circulating design leads to the gradual accumulation of copper over time, raising potential concerns related to health, surface staining, and material corrosion.^23,24^ Second, in addition to the primary inactivation occurring within the LEEFT-Cu device, the residual copper accumulated in the reservoir provides a secondary and continuous disinfection, altering the disinfection dynamics of the system. However, research on the performance and safety of LEEFT-Cu within circulating systems remains limited, constraining its practical application in recreational water environments.

In this study, we integrated a LEEFT-Cu device into a bench-scale circulating system to simulate the in-line disinfection processes of recreational water environments. The effects of current and flow rate on copper accumulation and microbial inactivation efficiency were systematically evaluated to elucidate the disinfection performance and operational stability of the system over time. Furthermore, real water samples were employed to assess the feasibility of LEEFT-Cu under realistic water conditions. The findings demonstrate the strong potential of LEEFT-Cu as an effective and sustainable in-line disinfection approach for circulating systems, such as swimming pools and hot tubs, thereby advancing the practical application of this emerging technology in recreational water management.

## 2 Materials and Methods

### 2.1 Chemicals and Materials

Luria–Bertani (LB) broth (Miller, cat# 97064) and LB agar (Miller, CulgeneTM, cat# 89405) were used for the growth and culture of bacteria. Sodium sulfate (Na_2_SO_4_, cat# 97062) was purchased from Avantor to adjust the conductivity of the water matrix. Copper sulfate (CuSO_4_, cat# 33308) was purchased from Alfa Aesar for the control experiments. Nitric acid (HNO_3_, 70%, cat# 225711) and a copper standard (TraceCERT, 1 g/L, cat# 38996) were purchased from Sigma-Aldrich for the atomic absorption spectrometer (PerkinElmer, PinAAcle 900F AA). CuVer1 Copper reagent powder pillows (Hach, cat# 2105869) were used for the UV–vis spectrophotometer (Hach, DR6000). Deionized water (18.2 MΩ·cm) was collected from a Thermo Scientific Barnstead Nanopure system for all the experiments and rinsing. *Escherichia coli* (*E. coli*, ATCC 10798) was used as the model bacterium for all the experiments.

### 2.2 Construction of the Circulating System

A bench-scale circulating system was developed to simulate recreational water environments (Figure S1). The system consisted of a LEEFT-Cu device, an acrylic tank, a power source (Keithley 2400 Sourcemeter), a peristaltic pump (MasterFlex L/S), and a mixer (IKA Eurostar 60 Digital). The tubing connecting the reservoir with the LEEFT-Cu device had an internal diameter of 3.1 mm and a length of 1 m, corresponding to a total loop volume of ∼7.5 mL. The LEEFT-Cu device was constructed following established procedures with slight modifications.^22^ Briefly, a stainless-steel tube (Amazon, 21.5 cm in length, 4.88 mm inner diameter) was positioned between two PVC blocks sealed with plugs, serving as the outer cathode. A copper wire (Arcor Electronics, 255 μm diameter) was suspended along the tube axis as the central anode. The resulting reactor chamber measured 24.5 cm in length with an effective volume of ∼5 mL. Electrodes were connected to the power source to enable ionization, and the copper wire was secured through the plugs to prevent short circuits. For system operation, a 10 L water sample was prepared and served as the reservoir (the reactor and loop volumes are included). The sample was circulated through the LEEFT-Cu device at a constant flow rate and returned to the reservoir. The reservoir was continuously stirred to ensure uniform distribution of copper and bacterial concentrations throughout the experiments.

### 2.3 Water Sample Preparation and Bacteria Culture

In the US, swimming pools and hot tubs are typically filled with municipal (tap) water that contains residual chlorine for secondary disinfection within distribution pipelines. In this study, to eliminate the influence of chlorine on microbial inactivation, real water samples were collected from the inlet of a local drinking water treatment plant to evaluate system performance under realistic water conditions. This raw water, which contains no chemical additives and is rich in NOM, was chosen to simulate the heavy organic load characteristic of recreational water environments. Considering the large experimental volume (*i.e.*, 10 L per test), synthetic water was also prepared for convenience by adding sodium sulfate (Na_2_SO_4_) to adjust the conductivity to match that of the raw water. The conductivity of all water samples was measured using an electrochemical meter (Orion Versa Star Pro). The parameters of the water samples are summarized in the Table S2.

*E. coli* was cultured aerobically in LB broth at 35 °C to a stationary growth phase, yielding a cell density of approximately 10^9^ CFU/mL. The culture was pelleted by centrifugation at 4000 rpm for 5 min. The supernatant was discarded, and the cells were resuspended in deionized water. This washing process was repeated three times to remove any residual growth medium and eliminate background effects from the broth. Subsequently, a 10 mL aliquot of the washed bacterial suspension was added to 10 L of synthetic or raw water samples, corresponding to a 1000-fold dilution. The inoculated solutions had a cell density of approximately 10^6^ CFU/mL and were used as the experimental reservoirs.

### 2.4 Disinfection Experiments and Copper Concentration Measurement

Bacterial concentrations were determined by the standard plate count (SPC) method, and copper concentrations were measured using an atomic absorption spectrometer (AAS).^25,26^ For the SPC method, samples were serially diluted by factors of 10, 100, and 1,000, and 100 μL of each dilution was uniformly plated onto agar plates. Under this scheme, the minimum detectable concentration is 1 colony forming unit (CFU) per 100 μL, equivalent to 10 CFU/mL. The inactivation efficiencies for the plug-flow and circulating systems were calculated using eqs 1 and 2, respectively

$$\mathrm{Inactivation}\;\mathrm{efficiency}=\log_{10}\frac{C_{\mathrm{influent}}}{C_{\mathrm{effluent}}}$$ 1

$$\mathrm{Inactivation}\;\mathrm{efficiency}=\log_{10}\frac{C_{0}}{C_{t}}$$ 2

where C_influent_ and C_effluent_ are the bacterial concentrations in the influent and effluent of the plug-flow system, respectively; C_0_ is the initial concentration in the reservoir at t = 0, and C_t_ is the concentration at sampling time t in the circulating system. Given that the initial inoculated bacterial concentration was approximately 10^6^ CFU/mL, the detection limit corresponds to a 5-log reduction. Experiments with the plug-flow system were conducted following previously reported protocols with minor modifications.^18^ Water samples were pumped through the reactor at flow rates ranging from 10 to 160 mL/min under constant current of 1, 2, or 4 mA. Constant current was applied instead of constant voltage to have a better control of copper ionization process.

For the bench-scale circulating system, water samples were collected directly from the reservoir at 30 min intervals. The reservoir volume was effectively constant throughout the experiments. Specifically, 10 mL of water was collected at the beginning and at each sampling time point for analysis. For a 4-h experiment, the total sampled volume was 90 mL, which is more than 2 orders of magnitude smaller than the 10 L sample and therefore has a negligible impact on copper and bacterial concentrations in the reservoir. Bacterial concentration, copper dosage, and pH were quantified by SPC, AAS, and the electrochemical meter, respectively, over the experimental period. To maximize the copper uptake by the bacteria, collected samples were held for 2 h before plating. Agar plates were incubated at 35°C for 14–16 h, after which visible colonies were enumerated. All SPC measurements were performed in triplicate, and each experimental condition was repeated in at least two independent trials to confirm reproducibility. All the experiments were conducted at room temperature (25 °C) without any evaporation control.

Copper disinfection experiments were conducted by introducing a concentrated CuSO_4_ solution directly into the reservoir in place of the LEEFT-Cu device. In this condition, microbial inactivation was achieved solely by copper exposure without the influence of the electric field (*i.e.*, no LEEFT). The CuSO_4_ concentration and dosing rate were adjusted to make copper accumulation comparable to that generated by the LEEFT-Cu device (Supplementary Note 1). Briefly, a 67 mg/L CuSO_4_ solution was continuously introduced into the reservoir at a constant flow rate of 0.5 mL/min. Copper concentrations during the disinfection experiments were monitored using CuVer1 copper reagent test kits (Hach Method 8506).

## 3 Results and Discussion

### 3.1 Efficacy of the Plug-Flow System for Practical Water Conditions

Previous LEEFT-Cu studies primarily used deionized water, which exhibits low conductivity (∼0.3 μS/cm), as the experimental matrix under relatively low flow rates (Table S1).^17,21,22^ To better simulate recreational water environments, the conductivity in this study was adjusted to ∼50 μS/cm, comparable to the conductivity of raw water (Table S2). Compared with deionized water, the higher ionic strength of this matrix reduced the system resistance, thereby increasing the current if applied voltage is fixed or decreasing the voltage when then current is constant, according to Ohm’s law.^27^ Neither of these two results desirable, because higher current causes excessive copper release in the effluent and high health concerns while lower voltage weakens the electric field and thus decreases inactivation efficiency. To solve this problem, we can increase the flow rate while maintaining the applied voltage, which preserves a strong electric field and simultaneously limits copper concentration in the effulent.^21^ High flow rates also enhance overall water circulation, which is generally preferred in pool and hot tub operations. Applied currents of 1, 2, and 4 mA corresponded to the voltages of 0.8, 1.3, and 2.2 V, respectively (Figure S2). These voltages were demonstrated in previous studies for effective microbial inactivation.^17^ Figure 1 shows the copper concentration and inactivation efficiency of a plug-flow system under various currents and flow rates. As shown in Figure 1a, the copper concentration decreases with increasing flow rate and decreasing current, which is expected according to Faraday’s laws of electrolysis (Supplementary Note 2). The actual copper concentrations are approximately 80% of the theoretical values (Figure S3), indicating high Coulombic efficiency and minor side reactions. It is worth noting that under some conditions (*e.g.*, low flow rate and high current), the copper releases are too high for plug-flow systems, but they can still be acceptable for circulating systems provided that the bulk copper concentration in the reservoir remains low. This part will be discussed in subsequent sections.

**Figure 1:**
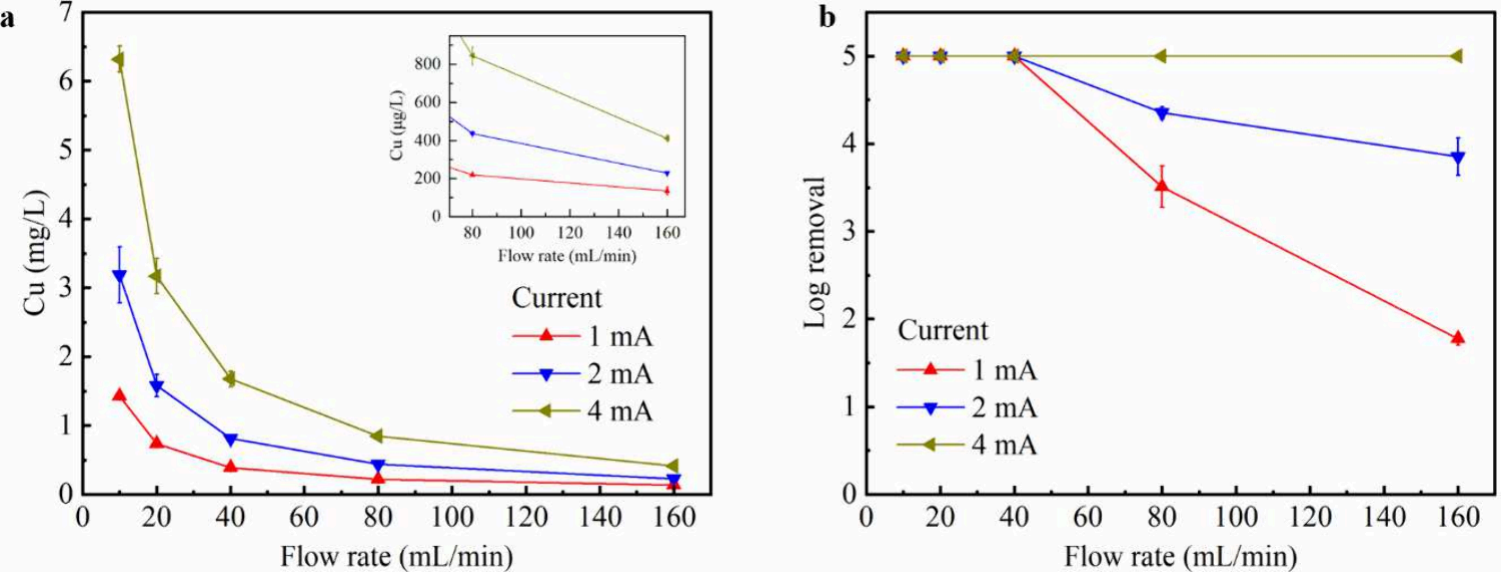
Performance of the plug-flow LEEFT-Cu system for practical water conditions. (a) Copper release and (b) log removal efficiency under different applied currents and flow rates. Error bars represent standard deviations of multiple trials and measurements.

As shown in Figure 1b, the log removal was maintained at 5-log (detection limited) when the flow rate was below 40 mL/min. At higher flow rates of 80 and 160 mL/min, 1 and 2 mA operation exhibited a decline in inactivation while 4 mA operation maintained a 5-log removal. These results are probably attributed to two factors. First, copper concentrations at low current and high flow conditions were insufficient for effective inactivation. For example, at 1 mA and 160 mL/min, the effluent copper concentration was only 135 μg/L (Figure 1a), lower than the reported antimicrobial threshold from previous LEEFT-Cu studies (*e.g.*, 200 to 500 μg/L).^17,18^ Second, hydraulic retention time (HRT) at high flow conditions was insufficient for bacteria to be transported to the center electrode, which is the hot spot for inactivation due to the locally enhanced electric field and in situ copper release.^17^ Interestingly, when the ratio of current to flow rate kept constant (*e.g.*, 2 mA, 80 mL/min vs 4 mA, 160 mL/min, or 1 mA, 80 mL/min vs 2 mA, 160 mL/min), copper concentrations remained at a similar level (Figure S4). Under these comparable conditions, high current exhibited a slightly higher inactivation than low current (Figure S4), indicating that electric field played a more vital role than HRT in determining disinfection performance. Overall, the LEEFT-Cu device maintained superior inactivation efficiency even under relatively high flow rates when treating the water with practical conductivity, showing great potential for practical water disinfection.

### 3.2 Effect of Current on the Circulating System

The previous section demonstrated that, in a plug-flow LEEFT-Cu system, copper release and microbial inactivation were influenced by both current and flow rate. However, the situation differs in a circulating system. First, since the circulation loop lacks influent and effluent streams, the copper released from the LEEFT-Cu device recirculates back into the reservoir, leading to gradual copper accumulation. Second, in addition to the direct inactivation occurring within the LEEFT-Cu device, the copper in the reservoir contributes to a secondary inactivation. As the system operates over time, the secondary inactivation from the accumulated copper in the reservoir becomes increasingly significant. Consequently, both copper and bacterial concentrations in the reservoir become functions of operation time, which is one of the key distinctions from the previous plug-flow systems. Considering that swimming pools typically require several hours to achieve complete chlorine mixing during refilling or concentration adjustment (system setup),^28^ an operation period of 4 h was selected in this study to monitor the temporal evolution of copper and bacterial concentrations within the circulating system.

Figure 2 shows the temporal profiles of copper concentration and microbial inactivation efficiency in the reservoir under various applied currents, with the flow rate fixed at 40 mL/min. As shown in Figure 2a, the copper concentration in the reservoir was proportional to the operation time. This result was not surprising because the copper release rate under constant current remained the same, resulting in a linear accumulation of copper over time (Supplementary Note 2). In addition, similar to the plug-flow system, copper concentration in the reservoir was proportional to the applied current (Figure S5). Notably, the copper accumulation at 4 mA was rapid, exceeding 800 μg/L after only 2 h. Consequently, the LEEFT-Cu device was turned off after 2 h, while the pump continued to operate for the remainder of the 4 h period. As mentioned in Section 3.1, for a circulating system, the key parameter of concern is the bulk copper concentration in the reservoir rather than instantaneous release from the device. For example, although the LEEFT-Cu device released copper at a rate of 1.68 mg/L under 4 mA and 40 mL/min (Figure 1a), the system could still operate safely for approximately 2 h before the reservoir copper concentration approached 1 mg/L. It is also worth mentioning that in a circulating LEEFT-Cu system, the LEEFT-Cu device is not designed for continuous operation. Instead, the LEEFT-Cu process functions as a setup phase to rapidly establish the target copper concentration and microbial inactivation in the reservoir (*e.g.*, 0.8 mg/L copper and 4-log removal). After the setup phase, operation mode would be switched to the LEEFT only (*i.e.*, using a stainless-steel center electrode without Cu release) to maintain the copper level in the reservoir. However, given that in practical recreational water environments, copper is continuously depleted due to swimmer exposure, filtration processes, and potential precipitation, the LEEFT-Cu device would be expected to operate intermittently or at a lower applied current to compensate for ongoing loss of copper.

**Figure 2:**
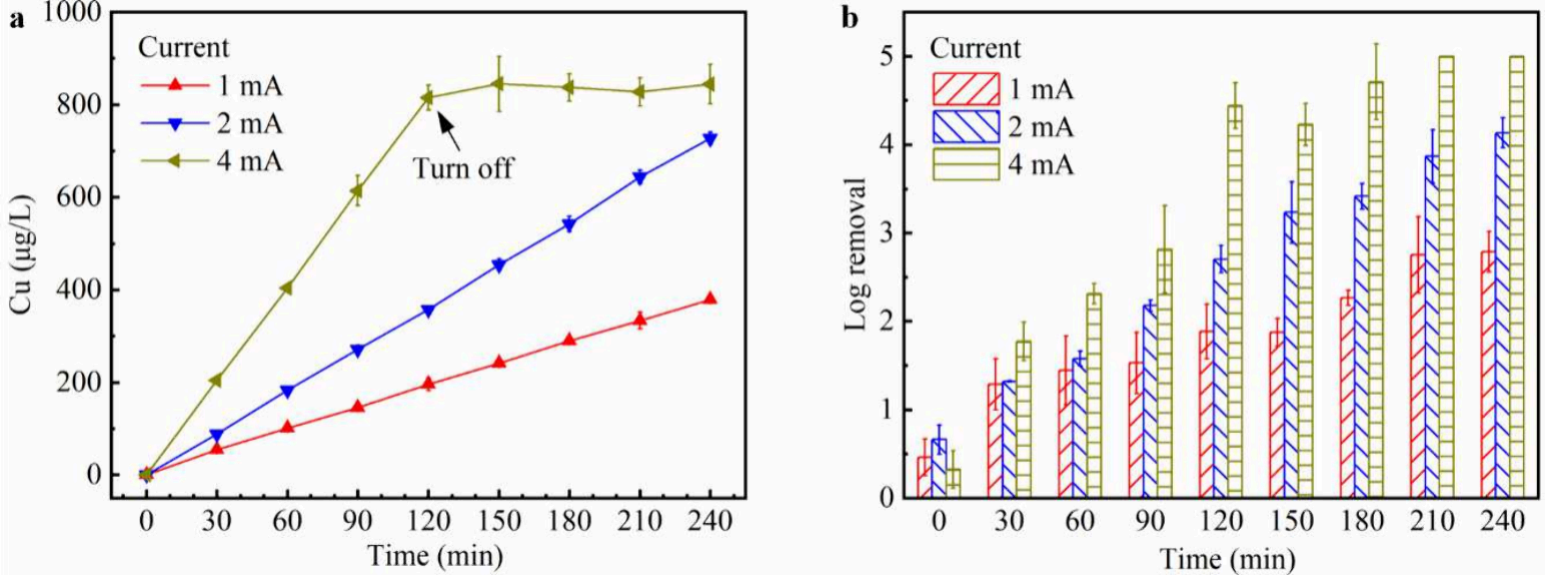
Performance of the circulating LEEFT-Cu system under different applied currents. (a) Copper concentration and (b) log removal efficiency in the reservoir over the operation time. The log removal was calculated relative to the initial bacterial concentration in the reservoir. Error bars represent standard deviations of multiple trials and measurements.

As shown in Figure 2b, the microbial inactivation efficiency in the reservoir increased with longer operation time and higher applied current. For example, at 4 mA, the inactivation exceeded 4-log after 2 h and continued to increase slightly after the device was turned off, whereas at 1 mA, the inactivation remained below 3-log even after 4 h. Notably, under the 4 mA condition, the slight increase in inactivation efficiency between 120 and 240 min (Figure 2b) indicated the residual disinfection by the accumulated copper in the reservoir and the absence of bacterial regrowth. Although a higher current led to a better disinfection performance, it also elevated copper concentrations in the reservoir (Figure 2a), which must be carefully controlled. In general, the inactivation efficiency is proportional to the copper concentration (Figure S6). Interestingly, when copper concentrations ranged from 200 to 600 μg/L, the inactivation efficiencies at 1 and 2 mA were higher than that those at 4 mA (Figure S6), likely due to the longer time required for low current to achieve the same copper level, allowing more water to be treated by the LEEFT-Cu device. Among the three currents tested in this study, 2 mA appeared optimal, achieving over 4-log removal within 4 h while maintaining a moderate reservoir copper concentration of 727 μg/L. Nevertheless, for practical applications, further optimization of the current and setup time in circulating systems will be necessary.

### 3.3 Effect of Flow Rate on the Circulating System

In addition to the applied current, flow rate is another parameter that may influence the performance of the circulating system. Based on the preliminary results, the flow rates of 40, 80, and 160 mL/min were selected, with the applied current fixed at 2 mA. The corresponding Reynolds number ranged from 196 to 784, indicating the laminar flow in the tubular reactor (Supplementary Note 3). Figure 3 shows the temporal profiles of copper concentration and microbial inactivation efficiency in the reservoir under these flow rates. The pH slightly increased from 5.6 to 5.9 over 4 h of operation and kept stable after the system was powered off (Figure S7). As shown in Figure 3a, the copper concentrations remained nearly identical across different flow rates, which contrasted with the results observed in the plug-flow device (Figure 1a**)**. This phenomenon occurs because under a constant current, the rate of copper release from the electrode remains unchanged, implying that the total copper introduced into the circulation is governed solely by the applied current and is independent of the flow rate (*i.e.*, how fast the water is circulated). This finding highlights another fundamental difference between plug-flow and circulating systems. In a plug-flow system, both current and flow rate influence copper concentration in the effluent, while in a circulating system, current is the dominant factor controlling overall copper concentration in the reservoir. Nevertheless, flow rate remains a critical design parameter in the practical operation of recreational water systems such as swimming pools and hot tubs. In this study, flow rates of 40–160 mL/min corresponded to turnover times of approximately 4–1 h. By comparison, recommended turnover times for residential swimming pools and hot tubs are 6 h and 30 min, respectively.^28,29^ These comparable time scales suggest that the bench-scale circulating system provides a relevant framework for extrapolation to real-world applications. For example, a 50,000 L swimming pool requires a circulation rate of 140 L/min to achieve a 6 h turnover time, which is 5,000 times the reservoir volume used in this study. Accordingly, the system flow rate can be increased by a similar factor (*e.g.*, from 40 mL/min at the bench scale to 200 L/min at full scale). To maintain comparable velocity and HRT within the LEEFT-Cu device, this increase can be accommodated by enlarging the reactor diameter and deploying multiple units in parallel (while adjusting the applied current to preserve comparable electric fields). Collectively, these considerations indicate that circulating LEEFT-Cu systems are amenable to rational scale-up and promising for practical applications in recreational waters with appropriate engineering design.

**Figure 3:**
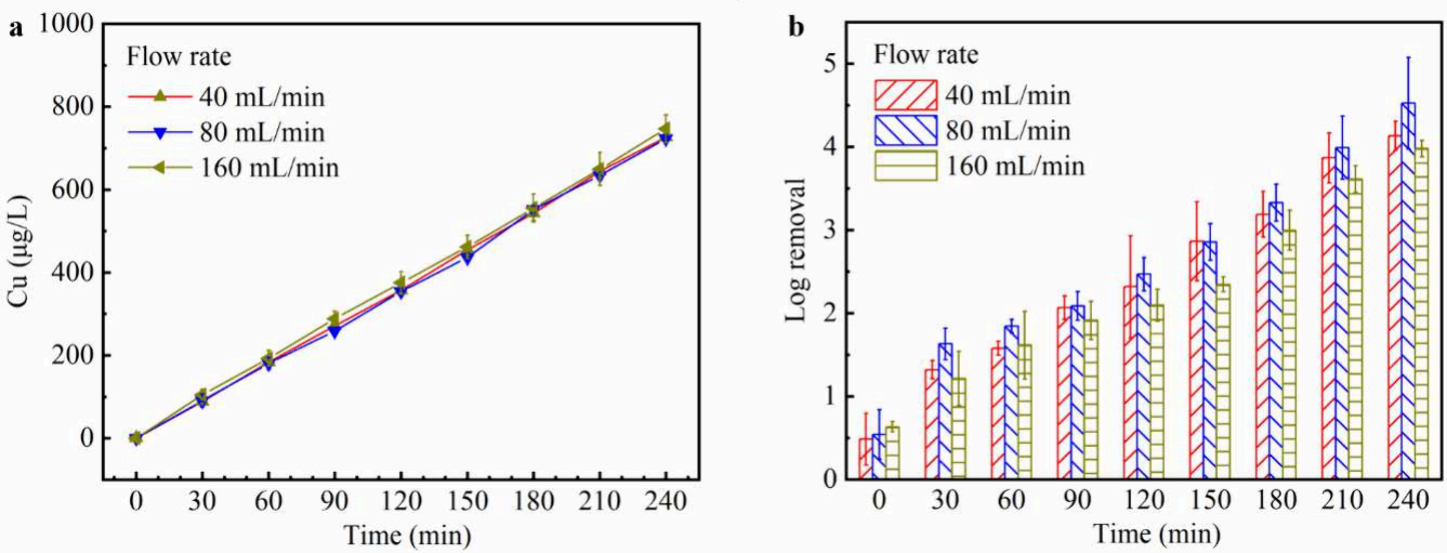
Performance of the circulating LEEFT-Cu system under different flow rates. (a) Copper concentration and (b) log removal efficiency in the reservoir over the operation time. The log removal was calculated relative to the initial bacterial concentration in the reservoir. Error bars represent standard deviations of multiple trials and measurements.

As shown in Figure 3b, the microbial inactivation efficiency in the reservoir exhibited an increase-to-decrease trend as the flow rates increased from 40 to 160 mL/min. This phenomenon is because a high flow rate implies a larger volume of water passes through the LEEFT-Cu device, which enhances overall inactivation efficiency. However, at excessively high flow rates, the HRT becomes sufficiently short that the exposure to the electric field is inadequate to significantly alter cell membrane permeability and facilitate copper entry. Under these conditions, the contribution of the electric field is diminished, and the inactivation is dominated by copper disinfection alone, thereby reducing disinfection efficacy of the LEEFT-Cu device (Figures 1b and 3b). Consequently, although the treated water volume increases, the proportion of bacteria inactivated per unit volume decreases, leading to lower overall inactivation efficiency in the reservoir. Therefore, a trade-off exists that excessively slow circulation could lead to delayed microbial inactivation, while overly fast circulation may compromise device efficacy. Among the three flow rates tested in this study, 80 mL/min yielded the best performance, achieving approximately 4.5-log removal within 4 h, compared to 4.1 and 3.9 logs at 40 and 160 mL/min, respectively. For practical applications, further optimization of the flow rate is needed to balance the system circulating rate with the efficacy of LEEFT-Cu device, maximizing disinfection performance of the whole circulating system. Notably, compared with previous plug-flow systems, although higher flow rates in this study weaken the bacterial transport toward the center electrode due to the shortened HRT, enhanced copper uptake by bacteria exposed to the electric field and elevated local copper concentrations due to in situ ionization remain applicable. In addition, the circulating system allows copper to accumulate in the reservoir over time, resulting in progressively increasing bulk copper concentrations entering the device. These factors likely explain why effective inactivation is still observed at relatively high flow rates (*e.g.*, >40 mL/min).

### 3.4 Comparison of Circulating LEEFT-Cu System with Cu Disinfection Alone

Antimicrobial metals have long been employed for controlling waterborne pathogens in swimming pools, hospitals, and even drinking water systems owing to their inherent biocidal properties.^31–33^ Among them, copper, silver, and zinc are the most commonly used, typically administered through Cu/Ag ionization units, Cu/Ag/Zn cartridges, or Cu-based algaecides.^13^ These conventional approaches rely on the dissolution of metal ions into water to provide residual disinfection. To benchmark the performance of the circulating LEEFT-Cu system against conventional Cu-based treatment, CuSO_4_ solutions were prepared to yield copper accumulation in the reservoir equivalent to that of the LEEFT-Cu system (Supplementary Note 1). As shown in Figure 4, the copper accumulation in the reservoir was adjusted to match that of the circulating LEEFT-Cu system operated at 2 mA, ensuring that the total copper exposure was comparable between the two systems. As discussed in Section 3.3, flow rate did not influence the copper concentration in the reservoir. Therefore, we selected the optimal conditions (*i.e.*, 2 mA and 80 mL/min) for comparison. As shown in Figure 4, despite similar copper accumulation, the inactivation efficiency of copper alone was significantly lower, reaching only 1.3-log removal after 4 h, whereas the circulating LEEFT-Cu system achieved over 4-log removal under the similar copper level. This pronounced difference highlights the crucial role of the LEEFT in facilitating microbial inactivation beyond the antimicrobial properties of copper alone.

**Figure 4:**
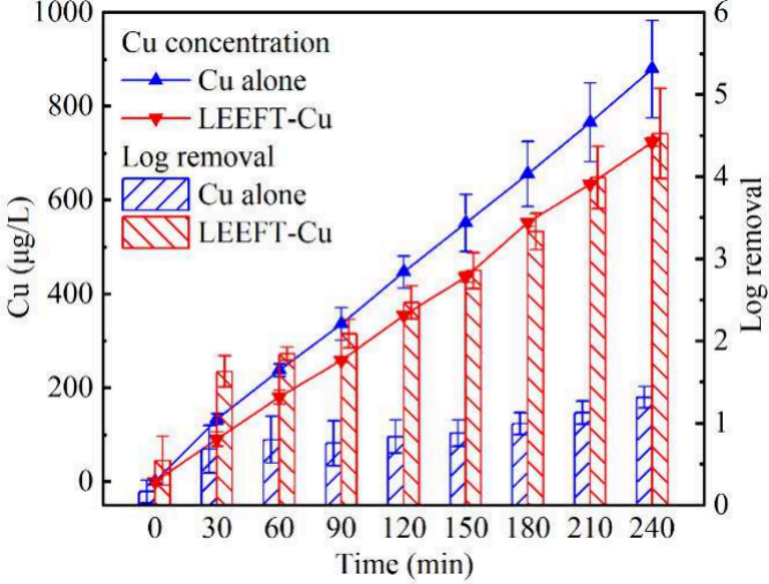
Performance comparison between the copper disinfection alone and the circulating LEEFT-Cu system (2 mA, 80 mL/min). The log removal was calculated relative to the initial bacterial concentration in the reservoir. Error bars represent standard deviations of multiple trials and measurements.

The observed limited disinfection efficacy of copper at low concentrations aligns with previous reports.^24,34,35^ The antimicrobial effects of copper primarily depends on its interaction with bacterial cell membranes, leading to oxidative stress and disruption of membrane integrity.^36–38^ However, such effects typically require high concentrations or prolonged exposure times. In contrast, the LEEFT-Cu system significantly enhances the antimicrobial potential through synergistic effects of electric field treatment and copper,^20^ which resulted in an approximately 3-log higher inactivation efficiency than CuSO_4_ dosing at the comparable copper concentrations (Figure 4). Collectively, these findings demonstrate that LEEFT-Cu achieves superior performance without additional chemical input, underscoring the potential of LEEFT-Cu to serve as a stand-alone, chlorine-free disinfection technology for circulating water systems.

### 3.5 Real-World Applications

To further evaluate the feasibility of LEEFT-Cu for real-world applications, experiments were performed using the real water samples collected from the inlet of a local drinking water treatment plant as the matrix. To eliminate the influence of chlorine on microbial inactivation, raw water, which contains no chemical additives and is rich in NOM, was chosen to simulate the heavy organic load characteristic of recreational water environments. The operational conditions were set at 2 mA and 80 mL/min, based on the results from Section 3.2 and 3.3. As shown in Figure 5, the temporal profile of copper accumulation in raw water was nearly identical to that observed in the synthetic water (Figure 3a), indicating that the presence of natural constituents did not significantly affect the copper release. However, the overall inactivation efficiency was slightly lower, achieving 3-log reduction after 2.5 h and 3.5-log reduction after 4 h (Figure 5). The moderate decline in efficacy may be attributed to the presence of NOM, suspended particles, and variations in pH and ionic strength, which influence copper bioavailability, antimicrobial efficacy, and precipitation behavior.^39^ In addition, some organic compounds may be oxidized within the LEEFT-Cu system and generate alternative nonchlorinated DBPs. Future work will focus on characterizing copper speciation and DBP formation, as well as evaluating LEEFT-Cu performance in waters with representative NOM levels to better assess system robustness under realistic operating conditions. Nonetheless, achieving over 3-log removal under realistic water conditions demonstrates that LEEFT-Cu remains highly effective for microbial control in recreational water environments such as swimming pools and hot tubs.

**Figure 5:**
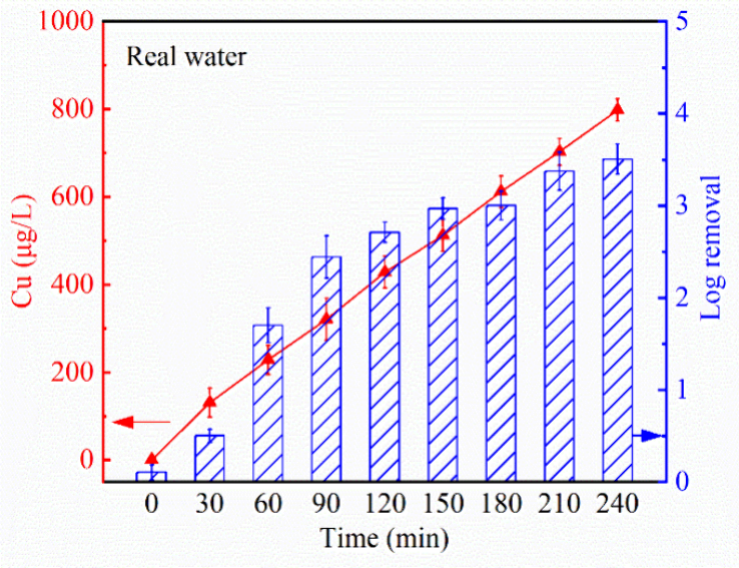
Performance of the circulating LEEFT-Cu system for the real water samples. The log removal was calculated relative to the initial bacterial concentration in the reservoir. Error bars represent standard deviations of multiple trials and measurements.

### 3.6 Additional Advantages, Limitations, and Future Work

The LEEFT-Cu system demonstrated strong potential as an energy-efficient and scalable in-line disinfection technology for circulating water systems, offering several distinct advantages. First, LEEFT-Cu operates effectively at low applied voltages. For instance, at an applied current of 2 mA and a flow rate of 80 mL/min, the corresponding voltage was only 1.27 V, with a power of 2.5 × 10^–3^ W (Figure S2). Besides, all components of the LEEFT-Cu device are commercially available and relatively low-cost materials, leading to low capital costs. Second, LEEFT-Cu provides advantages over conventional disinfectants. The copper accumulated in the reservoir offers residual disinfection effects, compensating for the lack of persistence typically associated with UV or ozonation systems.^22^ In contrast to volatile chlorine or chloramines, copper is more convenient for transport and storage and eliminates the concerns over respiratory health effects.^22^ Moreover, given that copper-based treatments such as Cu/Ag ionization units, Cu/Ag/Zn cartridges, and Cu-based algaecides have already been employed in swimming pool management, the LEEFT-Cu system can serve as a functional replacement for direct Cu ion dosing, providing both microbial and algal control while avoiding the operational issues associated with chemical addition.^13,40–42^

Despite these advantages, several challenges remain before LEEFT-Cu can be fully implemented at a practical scale. First, this study only explored a limited range of operational conditions (three currents and three flow rates) and used *E. coli* as the only test microorganism to demonstrate the proof of concept for LEEFT-Cu feasibility in circulating systems. Future work should systematically optimize operational parameters, including current, flow rate, and setup duration, to balance disinfection efficacy and copper accumulation for the different volumes of pools or hot tubs. Additional representative pathogens relevant to recreational waters (*e.g.*, *Pseudomonas* and *Legionella*), along with advanced analytical techniques (*e.g.*, flow cytometry and qPCR), will be employed to further evaluate system robustness under real-world conditions and to assess viable but nonculturable or sublethally injured bacteria. It is also worth mentioning that our group has previously explored asymmetric electric pulses to decouple electric fields with copper release, improving the device performance under the comparable flow rate, which can be adapted to the circulating system.^18^ Second, the stability of copper under varying environmental conditions should be carefully evaluated. Elevated temperatures and alkaline pH can promote copper precipitation, potentially leading to surface staining or fouling of plumbing components.^23,43^ It is also important to consider the regulatory and aesthetic constraints associated with copper in recreational water. Although the USEPA established the maximum contamination level goal for copper in drinking water as 1.3 mg/L to prevent gastrointestinal distress and protect against chronic exposure through ingestion,^44^ lower limits are often recommended for recreational use to avoid staining, corrosion, and potential skin or eye irritation. For instance, the World Health Organization’s Guidelines for Safe Recreational Water Environments suggest maintaining copper concentrations below 1 mg/L, whereas the Pool and Hot Tub Alliance recommend an operational range of 0.2–0.4 mg/L.^45,46^ These considerations highlight the need for future optimization of the LEEFT-Cu system to achieve effective disinfection at lower copper concentrations, thereby ensuring both public health safety and aesthetic water quality.

Lastly, in recreational water environments, copper and bacterial concentration in the reservoir is expected to fluctuate due to removal by filtration systems, adsorption onto pool surfaces, or loss through swimmer contact. An automated feedback control system capable of monitoring real-time copper levels and dynamically adjusting the applied current would be highly beneficial. Specifically, an inline copper sensor could be used to continuously measure the copper concentration in the reservoir, with the sensor output linked to the power supply through a control interface (*e.g.*, PC, microcontroller, or smartphone-based system). Based on a predefined target concentration range (*e.g.*, 400–800 μg/L), the applied current could be automatically adjusted, either through on/off control or variable-current operation, to compensate for copper loss and prevent excessive accumulation. This closed-loop control strategy would enable stable maintenance of residual copper concentrations within recommended limits. Potential bacterial loss due to attachment to system surfaces or other physical removal mechanisms will also be a crucial factor to be investigated in future pilot-scale or field studies. Collectively, these improvements will be essential for translating LEEFT-Cu from a bench-scale proof of concept into a practical, chlorine-free solution for recreational water disinfection.

## 4 Conclusions

In this study, we integrated a LEEFT-Cu device into a 10 L circulating system to simulate in-line disinfection processes in recreational water environments. The plug-flow LEEFT-Cu system maintained high efficacy under relatively high flow rates when treating the water with a practical conductivity (∼50 μS/cm). For the circulating system, the effects of current and flow rate on copper accumulation kinetics and microbial inactivation, including both primary inactivation within the device and secondary inactivation by the accumulated copper in the reservoir, were systematically assessed. Increasing current accelerated copper accumulation and shortened setup time, whereas lower current enabled higher microbial inactivation under comparable copper exposure due to more water directly treated by LEEFT-Cu. In contrast, flow rate primarily influenced microbial inactivation rather than copper accumulation. Although higher circulation enhanced treatment throughput, excessively high flow hindered bacterial exposure to localized electric fields, thereby reducing overall inactivation efficiency. Therefore, there are trade-offs for both current and flow rate selection, which requires further optimization. Among the conditions tested in this study, 2 mA and 80 mL/min demonstrated the best performance, achieving a 4.5-log removal at a copper concentration of 724 μg/L with power of 2.5 × 10^–3^ W. Compared to copper disinfection alone, the circulating LEEFT-Cu system exhibited an approximately 3-log higher inactivation, highlighting LEEFT-Cu as a stand-alone disinfection technology. Furthermore, the system maintained stable copper release with slightly decreased microbial inactivation under realistic water conditions, underscoring its scalability and promise as an energy-efficient, chlorine-free strategy for sustainable management of recreational water environments.

## Supplementary Material



## Data Availability

Data will be made available on request.

## Notes

Copper disinfection experiments and calculations of copper concentration and Reynolds number, review of current LEEFT-Cu studies and the parameters of water samples, and experimental setup and data analysis of the experiments (PDF)
