# Supplementary material for: Combined Locally Enhanced Electric Field Treatment and Copper for Effective Disinfection in a Circulating Water System
Source: ACS ES T Eng. 2026 Mar 5;6(4):1307–15. doi: 10.1021/acsestengg.5c01041 (PMC13077633; doi:10.1021/acsestengg.5c01041)
Supplement: Supplementary file 1 [file ee5c01041_si_001.pdf]

Supporting information for

Combined Locally Enhanced Electric Field Treatment and Copper for Effective Disinfection in a Circulating Water System

Feiyang Mo[1], Wei Wang[1], Lavine M Chuol[1], Mourin Jarin[1], James Willie Corley[1,2], Xing Xie[1,*]

[1] School of Civil and Environmental Engineering, Georgia Institute of Technology, 311 Ferst Drive, Atlanta, GA, 30332, United States

[2] Tift College of Education, Mercer University, 3001 Mercer University Drive, Atlanta, GA, 30341, United States

* Corresponding author(s): xing.xie@ce.gatech.edu

This file includes:

Supplementary Notes 1 - 3

Supplementary Tables S1 - 2

Supplementary Figures S1 – S7

Supplementary References

Supplementary Notes

1. Copper disinfection experiments

Copper disinfection experiments were conducted by introducing a concentrated $CuSO_4$ solution directly into the reservoir in place of the LEEFT-Cu device. In this condition, bacterial inactivation was achieved solely by copper exposure without the influence of the electric field (*i.e.*, No LEEFT). To maintain the copper concentration in the reservoir at a similar level to the circulating LEEFT-Cu system, the $CuSO_4$ concentration and dosing rate were adjusted. First, a low flow rate of 0.5 mL/min was selected to avoid significant volume change of the reservoir (10 L). Based on the results of disinfection experiments, the objective was to achieve 800 μg/L of copper within 4 hours. Then, the $CuSO_4$ concentration can be calculated as follows.

$$\text{Total copper} = 800\,\frac{\mu g}{L} \times 10\ L \times \frac{1\ mg}{1000\ \mu g} = 8\ mg$$

$$\text{Copper dosing rate} = \frac{8\ mg}{4\ h} \times \frac{1\ h}{60\ min} = \frac{1}{30}\ mg/min$$

$$CuSO_4\ \text{concentration} = \frac{1\ mg}{30\ min} \div 0.5\,\frac{mL}{min} \times \frac{1000\ mL}{1\ L} = 67\ mg/L$$

2. Theoretic calculation of copper concentration

According to Faraday's laws of electrolysis, at a constant current, the theoretic copper releasing rate from a single LEEFT-Cu device can be calculated using Eqn. S1.

$$r = \frac{m}{t} = \frac{MQ}{tnF} = \frac{MI}{nF} \quad (S1)$$

where r is the releasing rate; m is the mass of the released copper; t is the operation time; M is the mole mass of copper which equals 64 g/mol; Q is the transferred charge; n is the number of transferred electrons per species which equals 2 for Cu^{2+}; F is the Faraday constant which equals 96,485 C/mol; I is the current.[1]

The copper concentration in the effluent of the device can be calculated using Eqn. S2.

$$[Cu^{2+}]_{effluent} = \frac{r}{J} = \frac{MI}{JnF} \text{ S2)}$$

where J is the flow rate. Eqn. S2 demonstrates that the effluent copper concentration of a single LEEFT-Cu device (plug-flow system) is determined by both current and flow rate and independent of the operation time. The coulombic efficiency of the device is calculated from the ratio of the measured to theoretic copper concentrations in the effluent, as shown in **Figure S2**.

The copper concentration in the reservoir can be calculated using Eqn. S3.

$$[Cu^{2+}]_{reservoir} = \frac{rt}{V} = \frac{MIt}{VnF} \text{(S3)}$$

where V is the volume of the reservoir. Eqn. S3 demonstrated that for a specific reservoir, the copper concentration in the reservoir (circulating system) is determined by operation time and current and independent of the flow rate.

3. Calculation of Reynolds number

Within the tubular reactor, the Reynold number can be expressed as Eqn. S4:

$$Re = \frac{QD}{\nu A} \text{(S4)}$$

where Q is the flow rate, m^3/s; D is the outer electrode diameter which equals 4.88×10^{-3} m; ν is the kinematic viscosity of the water which equals 0.89×10^{-3} m^2/s at 25°C; and A is the cross section area of the reactor which equals 1.865×10^{-5} m^2. When flow rates ranged from 40 to 160 mL/min (*i.e.,* 6.67×10^{-7} to 2.67×10^{-6} m^3/s), the Reynolds number ranges from 196 to 784, indicating the laminar flow in the tubular reactor.

Table S1. A review of LEEFT-Cu studies

System	Water conductivity (μS/cm)	Voltage (V)	Flow rate (mL/min)
Plug flow reactor[2]	~0.3 (deionized water)	1.5 (DC)	2-20

Plug flow reactor[3]	~0.3 (deionized water)	1.5 (DC)	2-10
Plug flow reactor[4]	~100 (Na_2SO_4 solution)	1-7 (pulses)	4.6
Plug flow reactor[5]	~0.3 (deionized water)	1.5 (DC)	1-5
Plug flow reactor[6]	Not mentioned (tap water)	0.2-3 (DC)	15.7-94.2
Circulating (This work)	~50 (Na_2SO_4 solution)	0.8-2.3(DC)	40-160

Table S2. The parameters of the synthetic and raw water samples

Parameters	Synthetic water	Raw water
pH	5.6	6.7
Conductivity (μS/cm)	48-51	59-64
Total organic carbon (TOC) (mg/L)	/	1.49*
Bromide (mg/L)	/	0.013*
Chloride (mg/L)	/	5.37*
Temperature (℃)	25	
Cu concentration (mg/L)	Nondetectable (detection limit ~0.01 mg/L)	

* Provided by the local water treatment plant

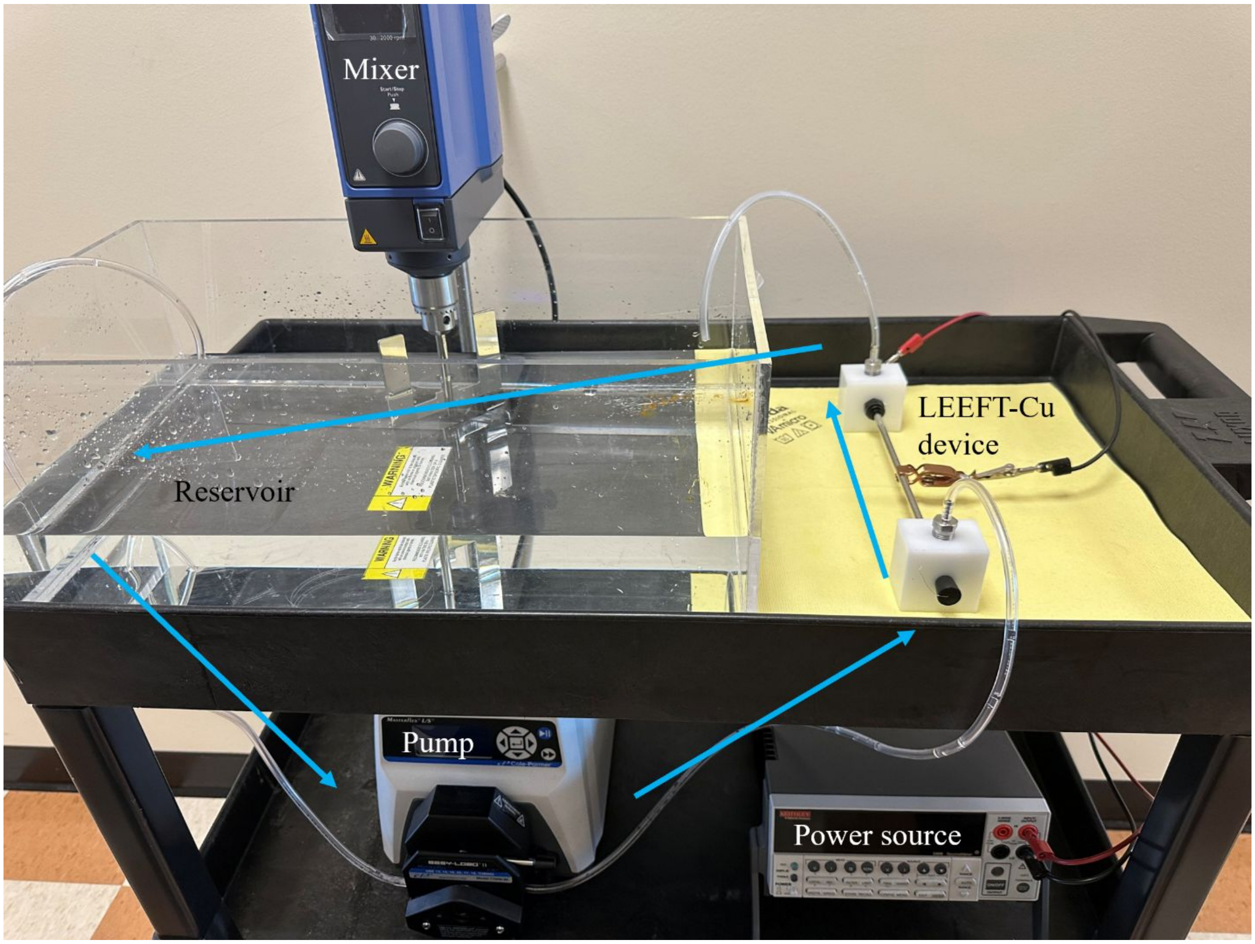

Figure S1. The circulating system. The blue arrows represent the water flow direction.

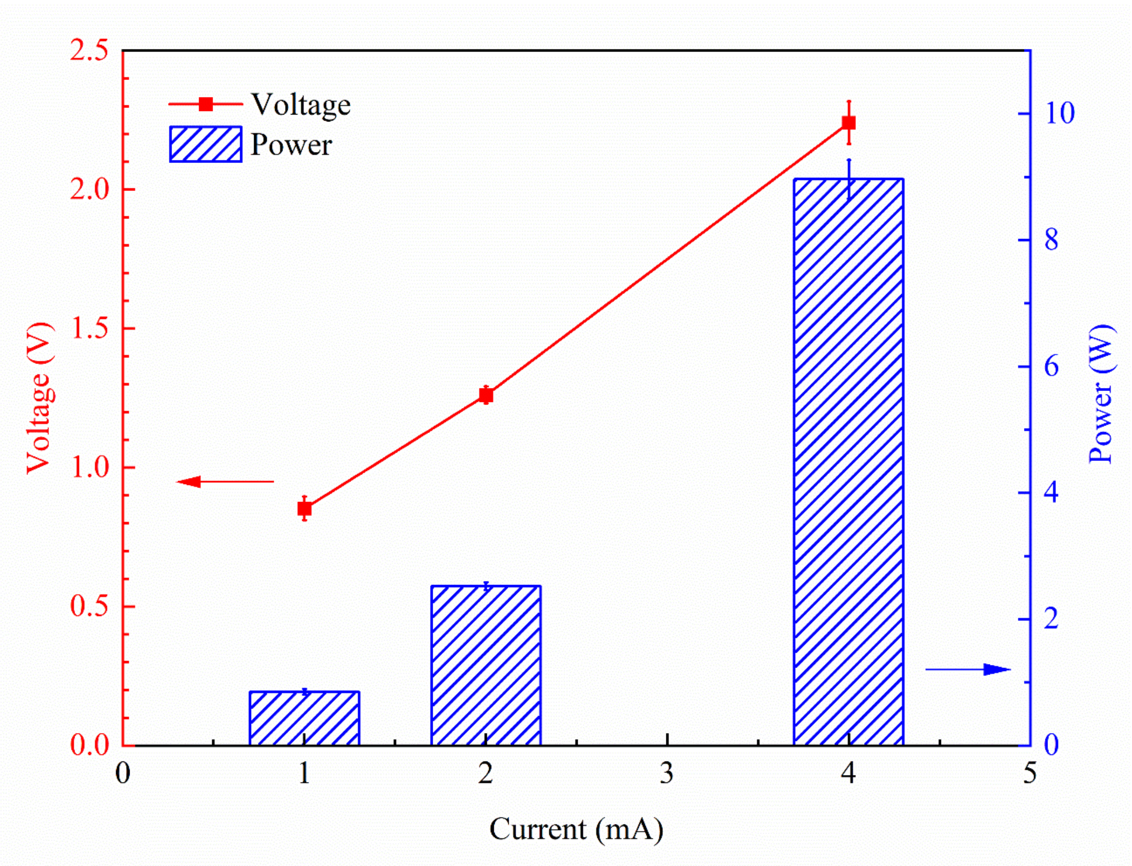

Figure S2. Voltages and powers of the circulating system under different currents. Flow rate did not affect the voltage and power at a constant current.

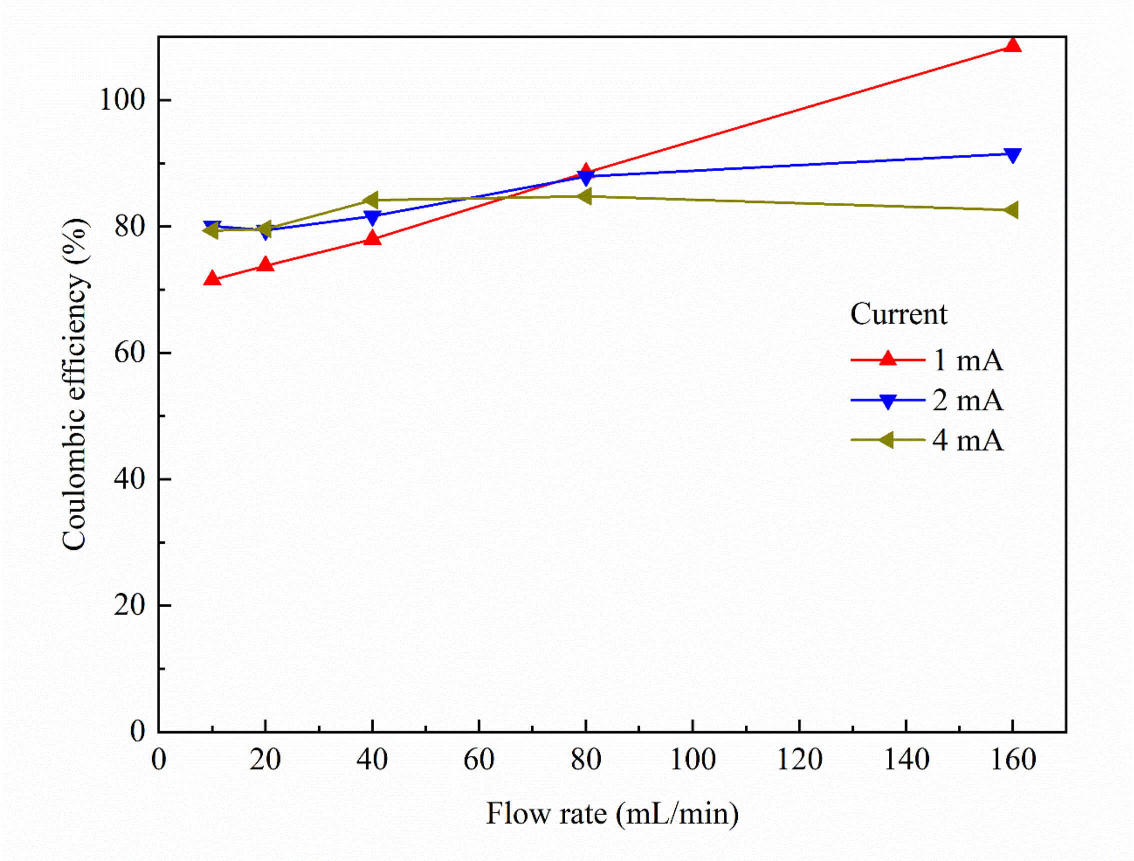

Figure S3. Coulombic efficiencies of the LEEFT-Cu device.

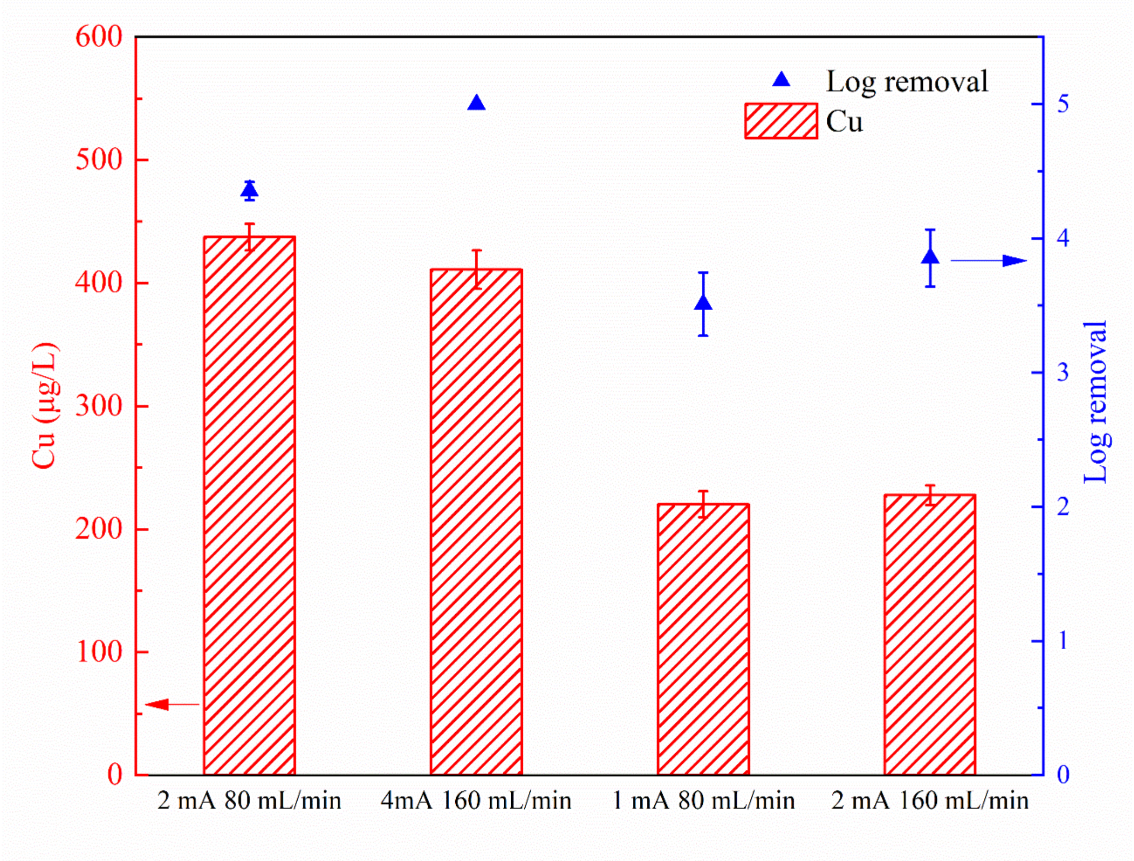

Figure S4. Comparison of inactivation efficiency under similar effluent copper concentrations in a plug-flow LEEFT-Cu system

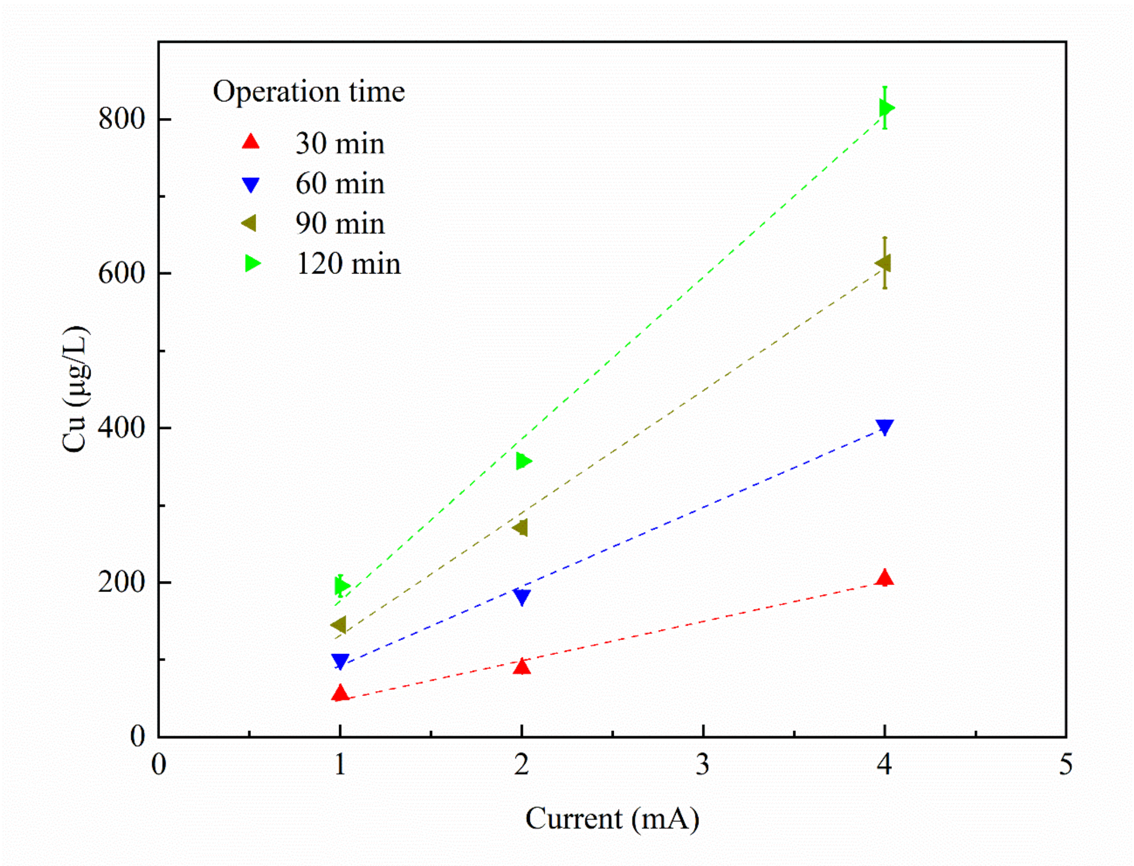

Figure S5. Copper concentrations in the reservoir under different currents along the time.

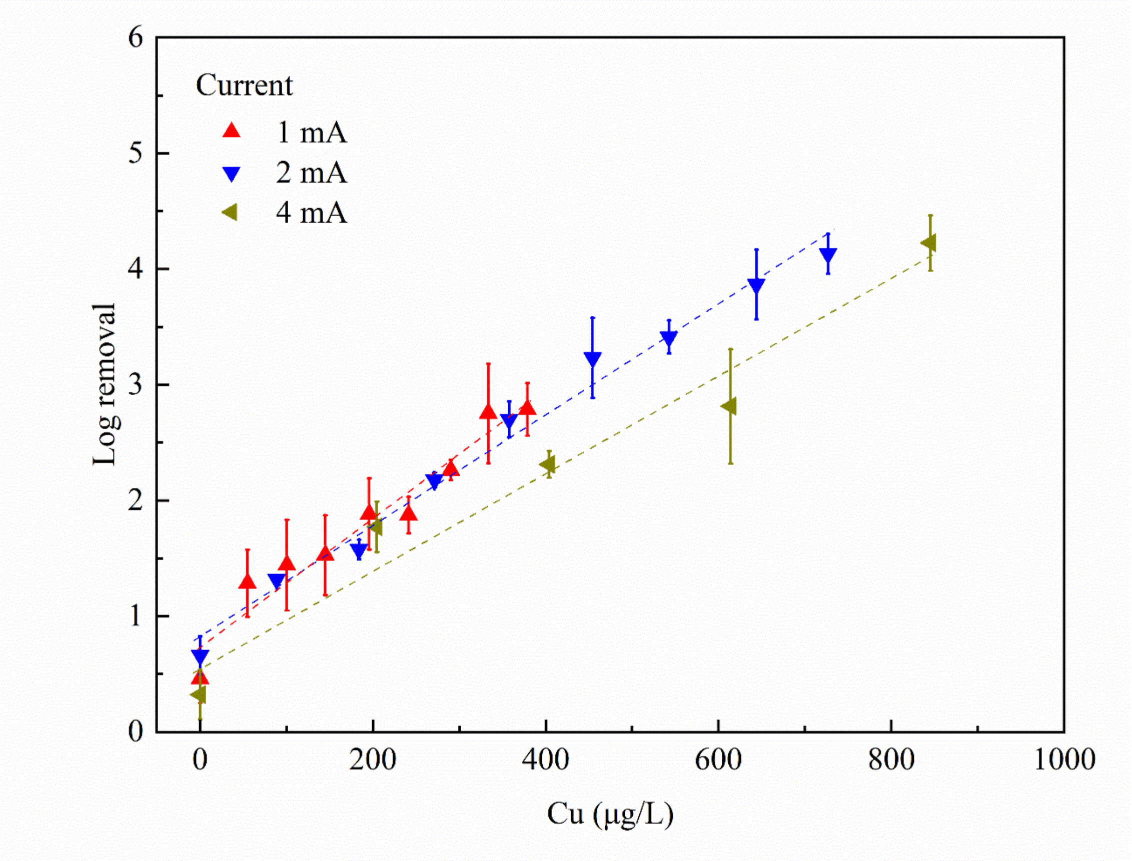

Figure S6. Relationship between inactivation efficiency and copper concentration in the reservoir.

Data collected after system shutdown is excluded for the 4 mA condition.

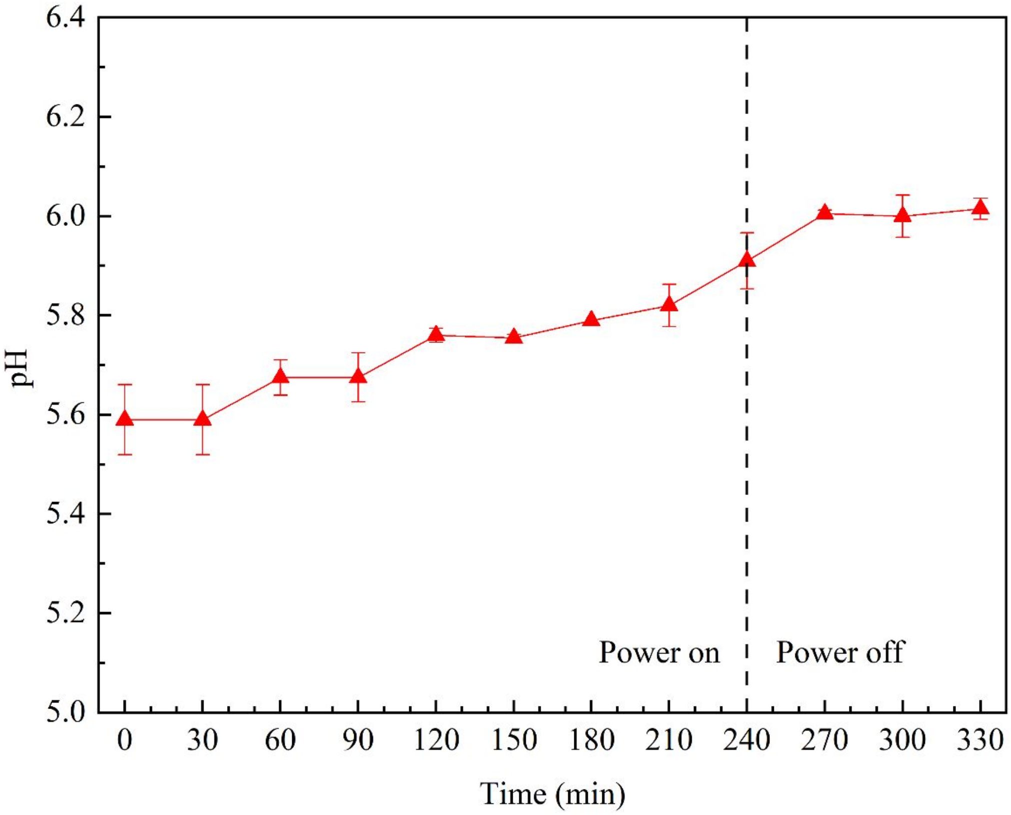

Figure S7. The pH variations over the experimental period. The applied current was 2 mA. Error bars represent standard deviations of the values observed under different flow rates.

Supplementary References

(1) Fuller, T. F.; Harb, J. N. *Electrochemical engineering*; John Wiley & Sons, 2018.

(2) Zhou, J.; Wang, T.; Xie, X. Rationally designed tubular coaxial-electrode copper ionization cells (CECICs) harnessing non-uniform electric field for efficient water disinfection. *Environment International* **2019**, *128*, 30-36. DOI: 10.1016/j.envint.2019.03.072.

(3) Zhou, J.; Yang, F.; Huang, Y.; Ding, W.; Xie, X. Smartphone-powered efficient water disinfection at the point of use. *npj Clean Water* **2020**, *3* (1), 40. DOI: 10.1038/s41545-020-00089-9.

(4) Mo, F.; Zhou, J.; Yu, C.; Liu, F.; Jumili, M.; Wu, Y.; Xie, X. Decoupling locally enhanced electric field treatment (LEEFT) intensity and copper release by applying asymmetric electric pulses for water disinfection. *Water Research X* **2023**, *21*, 100206. DOI: 10.1016/j.wroa.2023.100206.

(5) Jarin, M.; Ly, J.; Crowley, A.; Liu, S.; Xie, X. Combined Locally Enhanced Electric Field Treatment and Copper for Effective Inactivation of Gram-Positive and Gram-Negative Bacteria in Water. *ACS ES&T Engineering* **2025**, *5* (11), 2833-2843. DOI: 10.1021/acsestengg.5c00309.

(6) Li, R.; Dai, H.; Wang, W.; Peng, R.; Yu, S.; Zhang, X.; Huo, Z.-Y.; Yuan, Q.; Luo, Y. Local Electric Field-Incorporated In-Situ Copper Ions Eliminating Pathogens and Antibiotic Resistance Genes in Drinking Water. *Antibiotics* **2024**, *13* (12), 1161. DOI: 10.3390/antibiotics13121161.
